# *In vivo* detection of ALFA-tagged proteins in *C. elegans* with a transgenic fluorescent nanobody

**DOI:** 10.17912/micropub.biology.001542

**Published:** 2025-04-18

**Authors:** Sophie Quintin, Maria Izabella Saad, Grégory Amann, Anne-Cécile Reymann

**Affiliations:** 1 Université de Strasbourg, IGBMC UMR 7104- UMR-S 1258, F-67400 Illkirch, France; 2 CNRS, UMR 7104, F-67400 Illkirch, France; 3 Inserm, UMR-S 1258, F-67400 Illkirch, France; 4 IGBMC, Institut de Génétique et de Biologie Moléculaire et Cellulaire, F-67400 Illkirch, France

## Abstract

To track tagged endogenous proteins *in vivo* , we created a *C. elegans* strain expressing a fluorescently-labelled nanobody directed against the ALFA-tag epitope. The strain, which expresses an anti-ALFA nanobody fused to mKate2, is healthy and allows clear detection of the ALFA-tagged junction protein DLG-1 at all stages. This method is adapted for live imaging, circumvents the need of immuno-histochemistry, and opens perspective to study protein function *in vivo* . The future detection of sensitive proteins can therefore be envisaged in nematodes by using transgenic nanobodies, or chromobodies, in combination with ALFA-tagging by CRISPR.

**Figure 1. Design of the CRISPR-Cas9 NbALFA-expressing C. elegans strain and in vivo validation with ALFA-tagged DLG-1:**
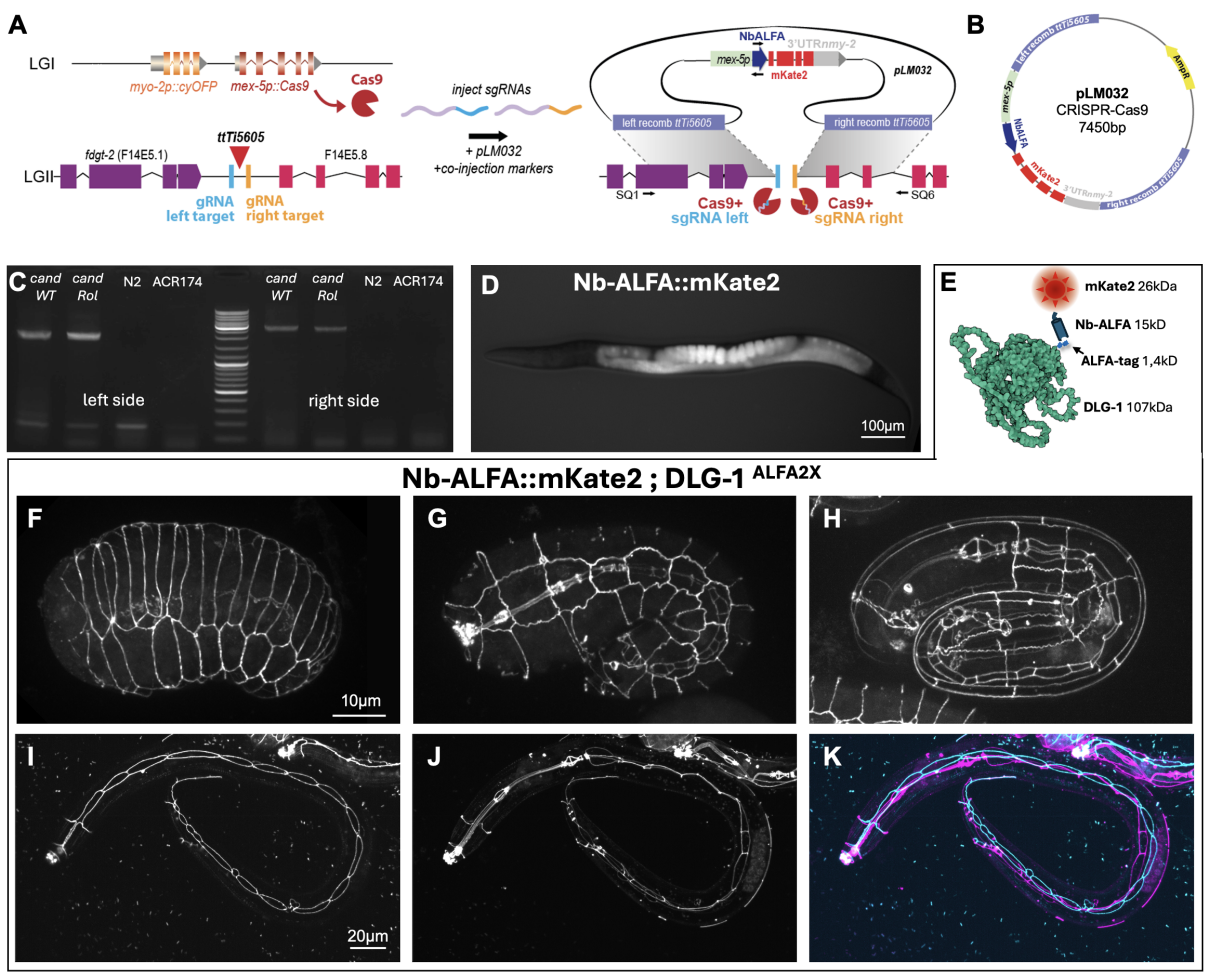
**A-** Design of the CRISPR-Cas9 strategy to target the *ttTi5605* locus. Left, starting strain ( ACR174 ) constitutively expressing Cas9 on chromosome I and showing the *ttTi5605* genomic region with the 2 gRNAs surrounding the Mos insertion locus on chromosome II. **B-** Schematic map of the pLM032 CRISPR-Cas9 vector to insert *mex-5 p* ::NbALFA at the MosSCI landing site *ttTi5605* on chromosome II. **C-** Gel picture of the PCR validating the insertion of pLM032 at the appropriate locus on chromosome II on each side (expected sizes 2182bp and 3000bp on left and right side, respectively), using primers depicted in A (also see Reagents, Primers for NbALFA insertion genotyping). *cand WT* and cand Rol indicate 2 F3 transgenics from which ACR194 was derived; N2 and ACR174 (starting strain) are negative controls. **D-** Red fluorescence coming from NbALFA-expressing worms ( ACR194 ) is detected at the fluorescence stereoscope. **E-** Schematic structure of ALFA-tagged DLG-1 (designed on Alphafold) bound by NbALFA::mKate2 **F-H-** Spinning-disc confocal projections of ACR206 embryos during elongation stages (lima bean F, 1.5-fold G, 3.5-fold H). The red fluorescence revealed by NbALFA is detected at adherens junctions in all tissues (epidermis, gut and pharynx), where DLG-1 ^ALFA^ is expressed. **I-K** - Spinning-disc confocal projections of an ACR206 L2 larva showing adherens junction labeling in the epidermis (I), along the digestive tract (J) or in the entire larva (K, combined projection of epidermal labeling in cyan and pharynx/gut labeling in magenta).

## Description

Protein fusion to fluorescent reporters can sometimes hinder protein function and cause some toxicity. In such cases, the introduction of short epitope tags is useful to detect proteins of interest. In addition, small tags circumvent the development of novel specific antibodies to each protein of interest. Recently, the short ALFA-tag epitope (only 13 amino-acids) has been developed, together with its cognate NbALFA nanobody for detection (Götzke et al. 2019). ALFA-tagging has been successfully applied in nematodes on two different proteins, DLG-1 and UNC-10 , whose detection was achieved by ALFA-tag immunostaining with commercial nanobodies on fixed animals (Igreja et al. 2022). Going a step further, we aimed at visualizing ALFA-tagged proteins in living animals by expressing a fluorescently-labelled transgenic nanobody, *ie* chromobody, as it has been reported in yeast (Akhuli et al. 2022).

We decided to insert the nanobody-coding sequence in a neutral genomic region and thus chose to target the frequently used *ttTi5605* MosSCI locus on chromosome II (Frøkjær-Jensen et al. 2008). We took advantage of an available MosSCI plasmid in our lab (Reymann et al. 2016) and fused the codon-optimized NbALFA DNA sequence to the codon-optimized sequence of a far red fluorophore (mKate2), under the control of the *mex-5* promoter and followed by the *nmy-2* 3'UTR, to generate the plasmid pLM031. A stretch of glycine and serine was introduced as a linker between NbALFA and mKate2 to favor correct protein folding. To bypass the need of injecting in the *unc-119 ( ed3 )* mutant background for MosSCI and to speed up the selection of edits, we sought to convert our plasmid in a CRISPR-Cas9 repair vector while keeping *ttTi5605* as a target site. We first selected two gRNA targeting this locus ( Figure 1A ). Then, we generated a new plasmid (pLM032, Figure 1B ), by shortening the left and right recombination sequences from pLM031 —to prevent Cas9 from cutting the repair template— and removed the *unc-119* rescuing element. Note that the cloning method employed to switch from the MosSCI to the CRISPR-Cas9 vector was a home-made version of commercial Gibson (or In-fusion) assembly kits, named SLiCE (Zhang, Werling, and Edelmann 2012), which proved to be highly successful at a very low cost (see Methods).

The pLM032 plasmid was injected along with gRNAs and co-injection markers in a wild-type looking strain constitutively expressing Cas9 ( ACR174 , adapted from Schwartz et al, 2021). The selection of edits relied on both PCR ( Figure 1C ) and screen for transgenic worms expressing mKate2 at the fluorescent dissecting scope. The expression of cytosolic and nuclear *mex-5* p::NbALFA::mKate2 was abundant and easily identifiable in gonads and early embryos ( Figure 1D ). We retrieved a single line matching the expected edited sequence, namely ACR194 , which was subsequently mated to the DLG-1 ::2xALFA endogenously tagged strain, carrying two tandem ALFA-tags inserted immediately upstream of the *dlg-1* stop codon (Igreja et al. 2022). The resulting strain ( ACR206 ) nicely showed red fluorescence at the levels of adherens junctions throughout embryonic ( Figure 1F- H) and post-embryonic development ( Figure 1I- K) in all epithelial cells where DLG-1 is expressed (Bossinger et al. 2001; McMahon et al. 2001). Interestingly, there was no residual fluorescence background coming from NbALFA alone including no nuclear signal, suggesting that it was not excessively expressed compared to its bait protein. Note that the *mex-5* p-driven expression of NbALFA::mKate2 allowed the detection of DLG-1 ^ALFA^ in a pattern not strictly restricted to the germline and early embryos as fluorescence is clearly observed in post-embryonic somatic cells. We hypothesize that there could be protein and/or mRNA perdurance in the lineage or a previously overlooked low *mex-5* p-driven expression in these cells. In addition, the use of a zygotic gene 3'UTR *nmy-2* may further contribute to reinforce long-term mRNA transgene stability. We conclude that NbALFA::mKate2 allowed accurate *in vivo* visualization of DLG-1 ^ALFA^ , similarly to what has been observed in fixed samples using a commercial nanobody (Igreja et al. 2022), or with a single copy integration of the DLG-1 ::RFP reporter (Diogon et al. 2007). Therefore, our work demonstrates that chomobodies can be successfully engineered in *C. elegans* .

We speculate that this combination ALFA-tag/NbALFA could be extended to study protein dynamics and function. Given its small size, the ALFA-tag can easily be inserted in any gene by CRISPR-Cas9, and is much less likely to affect protein function compared to a classical reporter fusion. Remarkably, nanobodies are single chain antibodies that not only enable protein visualization *in vivo* , but also allow protein manipulation *in vivo* and biochemical studies (reviewed in (Helma et al. 2015). For example, nanobodies were engineered to alter protein subcellular localization in a so-called Nanotrap system in Drosophila (Xu et al. 2022). In *C. elegans* , nanobody-mediated degradation can be achieved for GFP-tagged proteins (Wang et al. 2017). Ultimately, our system is highly versatile; for example, the NbALFA can be driven by a tissue-specific promoter to focus on cells of interest. In addition, the nanobody can be coupled to any other fluorophore to facilitate multicolor observations with existing tagged strains, which avoids to introduce different reporters by CRISPR. Therefore, we speculate that this ALFA-tag/NbALFA system constitutes a valuable tool with a high potential for future applications, which adds to the existing toolbox of *C. elegans* .

## Methods

**Plasmid construction**

A g-block encoding the anti-ALFA-tag nanobody (from Addgene plasmid n°159986), with a C-terminal GSGSGS linker and codon-optimized for *C. elegans* (Redemann et al. 2011) was ordered at IDT. The NbALFA was cloned into the MosSCI vector pLM025 (Reymann et al. 2016) between NotI and AscI restriction sites, in frame with mKate2. A digestion with the restriction enzyme NheI was done prior to transformation in DH5alpha competent cells in order to eliminate empty vectors clones. After insert detection by restriction on minipreps (Macherey Nagel miniprep kit), only one out of 4 positive clones tested did not carry any mutation and was further validated by whole plasmid sequencing (Eurofins), giving rise to pLM031. To convert this MosSCI plasmid into a CRISPR-Cas9 vector, we got rid of undesired sequences (shortening LRS and RRS and removing *unc-119* rescuing element) by a double PCR strategy, using primers designed with a 15bp overlap for subsequent plasmid assembly (sequences below). The PCR was run on pLM031 template using CloneAmp HiFi PCR Premix (Takara), according to the manufacturer's recommendations ( *ie* 98°C for 10s, 55°C for 15s, 72°C for 25s, repeated 35 cycles, with an initial 3 min denaturation step of 98°C and a final 3 min elongation step). A DpnI digestion was performed (1h at 37°C) to remove plasmid template. Each PCR product was gel-purified for the subsequent SLiCE reaction, a home-made version of commercial Gibson or In-Fusion cloning kits (Zhang et al 2012), as described here (Okegawa and Motohashi 2015). After transformation and selection of positive clones, pLM032 was validated by whole plasmid sequencing (Eurofins).

**Transgenesis**

*C. elegans* strains were maintained at 20°C according to standard methods (Brenner 1974). An injection mix composed of left and right sgRNAs (crRNAs and tracRNA ordered at IDT), pLM032, pRF4 and fluorescent co-injection markers was injected in ACR174 , according to (Schwartz et al. 2021). Transgenics were isolated and screened by PCR (Accustart kit, QANTA Bioscience) for plasmid insertion in the genome at the *ttTi5605* locus (see Reagents, Primers for NbALFA insertion genotyping) as well as by detection of fluorescence using a stereo microscope (Zeiss Discovery.V20), but only one insertion was recovered ( Figure 1C, D). The Cas9 transgene was removed by outcrossing with N2 , generating the ACR194 strain, which was mated to RS4103 .

**Image acquisition**

For live imaging, embryos were mounted between slide and coverslip on 3% agarose pads in M9; 1mM levamisole was added for animal observations. Spinning-disk confocal imaging was performed on an inverted DMI8 Leica microscope equipped with a Yokogawa CSUW1 head, an Orca Flash 4.0 camera piloted by the Metamorph software. Objective used were oil-immersion 40X and 100X. The temperature of the microscopy room was maintained at 20˚C. Z-stacks of 20 to 30 images were acquired every µm across the whole embryo or larva. Maximum intensity projections were performed using the Fiji software and used to generate the images shown.

## Reagents

**Strains**

**N2** Bristol reference strain

**ACR174** W01A8.6 ( *oxTi1128* [ *mex-5 p* ::Cas9(+ *smu-2* introns), *hsp-16.41* ::Cre, *myo-2p* ::2XNLS-CyOFP + lox2272]) I obtained after removing *unc-119 ( ed3 )* from the background of **EG9887** (Schwartz et al. 2021).

**ACR194** *reySi01* [ *mex-5 p* ::NbALFA:: *mKate2* _ *nmy-2* 3'UTR] II

**ACR206** *reySi01* [ *mex-5 p* ::NbALFA:: *mKate2* ] II; *dlg-1* ( *tu1782* [ *dlg-1 :* :2XALFA]) X obtained after mating ACR194 with **RS4103** (Igreja et al. 2022).

**guideRNAs sequences targeting *ttTi5605***

**MosSCI_LRS_Left_gRNA (PAM in bold, not included):**

AAGTGAGTTTGCTACCATCA **TGG**

**MosSCI_RRS_Right_gRNA (PAM in bold, not included):**

GATATCAGTCTGTTTCGTAA **CGG**

**Primer sequences for MosSCI to CRISPR-Cas9 conversion**

Primers for first amplicon corresponding to vector backbone with shorter LRS and RRS sequences, without *unc-119* rescuing element

**oAC115 sens** (overlapping **3'UTR** & RRS) **taaCGGtcttctgta** taactacaaaaaagaataaaaaaccgtatc

**oAC116 rev** (overlapping ***mex-5* p** & LRS) **tcaTGGaatcaggag** gacatttcgacaatgtc

Primers for second amplicon containing *mex-5 p* ::NbALFA:: *mKate2* _3'UTR *nmy-2*

**oAC117 sens** (overlapping **LRS** & *mex-5* p): **ctcctgattCCAtga** TATCCTGCAGGAATTCCTCGAGGTC

**oAC118 rev** (overlapping **RRS** & 3'UTR) **tacagaagaCCGtta** GGGCAGATCTgatagcggttgtttg

**Primers for NbALFA insertion genotyping**

**Left side integration checking**

**SQ1 sens** (outer left recombination sequence, in the genome) GGCAGAATGTGAACAAGACTC

**oAC114** **rev** (in NbALFA) GGGATCCTCCTGGTTGGACG

**Right side integration checking**

**oAC113** **sens** (in NbALFA) GGGGACAAGGAACCCAAGTC

**SQ6 rev** (outer right recombination sequence, in the genome) GCGAACCTAACTGTAAAAGTCC
